# Generative Adversarial Networks-Based Semi-Supervised Automatic Modulation Recognition for Cognitive Radio Networks

**DOI:** 10.3390/s18113913

**Published:** 2018-11-13

**Authors:** Mingxuan Li, Ou Li, Guangyi Liu, Ce Zhang

**Affiliations:** National Digital Switching System Engineering and Technology R&D Center, Zhengzhou 450001, China; seu_lmx@foxmail.com (M.L.); zzliou@163.com (O.L.); cezhang@foxmail.com (C.Z.)

**Keywords:** automatic modulation recognition, deep learning, semi-supervised, generative adversarial network

## Abstract

With the recently explosive growth of deep learning, automatic modulation recognition has undergone rapid development. Most of the newly proposed methods are dependent on large numbers of labeled samples. We are committed to using fewer labeled samples to perform automatic modulation recognition in the cognitive radio domain. Here, a semi-supervised learning method based on adversarial training is proposed which is called signal classifier generative adversarial network. Most of the prior methods based on this technology involve computer vision applications. However, we improve the existing network structure of a generative adversarial network by adding the encoder network and a signal spatial transform module, allowing our framework to address radio signal processing tasks more efficiently. These two technical improvements effectively avoid nonconvergence and mode collapse problems caused by the complexity of the radio signals. The results of simulations show that compared with well-known deep learning methods, our method improves the classification accuracy on a synthetic radio frequency dataset by 0.1% to 12%. In addition, we verify the advantages of our method in a semi-supervised scenario and obtain a significant increase in accuracy compared with traditional semi-supervised learning methods.

## 1. Introduction

Currently, due to fixed spectrum allocations and expanding numbers of wireless devices, spectrum resources are becoming increasingly scarce. Therefore, researchers’ interests have shifted to improving opportunistic spectrum access techniques, also known as cognitive radio (CR) [1,2]. CR emphasizes the ability to learn, meaning that CR can interact with its local communication environment and change its own transmission parameters based on the interaction. The ability to perceive the spectrum environment is called spectrum sensing [3]. Spectrum sensing is the basic function of cognitive radio systems. It is a prerequisite for dynamic spectrum management and spectrum sharing. Spectrum resources can be used solely based on environmental awareness and detection. A key enabler in spectrum sensing is automatic modulation recognition (AMR), which is also essential in spectrum interference monitoring, radio fault detection, and a wide variety of civilian and military applications. For many years, AMR has been accomplished by carefully hand-crafting specialized feature extractors for different signal types. These feature-based methods [4,5,6] achieve good performance in certain scenarios, but their results are achieved through expert manual work. Thus, it limits the ability of CR system to perform adaption to unknown tasks and signals. Instead, automatic feature-learning methods that do not rely on human experience need to be applied. These methods should have the ability to recognize signal from ground-up features.

Recent breakthroughs in fields such as natural language processing (NLP) [7], speech signal processing [8] and computer vision [9] (CV) have been made with the aid of deep learning (DL). With the performance improvement of graphics processing unit (GPU) and parallel computing, computing cost of neural network (NN) has been cheaper. DL can greatly benefit from this technological advancement. Automatic feature extraction is one of DL’s foremost abilities: DL feature extraction methods can outperform those that rely on features designed by experts. Researchers are enthusiastically applying automatic feature extraction ability to the signal processing domain. We will introduce some typical results in the next section.

In this paper, we introduce the generative adversarial network (GAN) into the radio machine learning domain. Modulation recognition are performed by a general, scalable, end-to-end framework. The concept of a GAN was first proposed by Goodfellow et al. [10] in 2014. A GAN consists of a generator network that produces samples from code, and a discriminator network that distinguishes real samples from fake ones. These two networks play a min-max game which improves the performance of both networks. GAN have been proven to be especially powerful tool in computer vision and image processing because of its ability to expand datasets for semi-supervised learning. We want to introduce this capability into the field of modulation identification.

This article introduces the end-to-end GANs framework into the field of modulation recognition. The main contributions of this paper are as follows:

- We provide a brief introduction to GANs, discuss their application in automatic modulation classification, and propose a suitable framework for this field.
- Based on the application characteristics of the cognitive radio field, a semi-supervised method is applied that makes full use of signal sampling. We compare our method based on GANs with the semi-supervised method used in traditional machine learning.
- We propose signal synchronization and normalization method based on the attention model. Using this module, the training stability of the whole framework is improved.
- The authenticity of the fake radio samples generated by the generator network is verified. A statistical analysis is applied to the original sampling and the learned model specifications. The learning ability of GANs’ on different modulation signals is verified.

The remainder of this paper is organized as follows: Section 2 presents related works. Section 3 introduces the basic concepts of GANs and the improved structural design for the spectrum sensing field. Section 4 introduces the methods and technical approaches for wireless signal classification. Section 5 describes experiments conducted on both a synthetic radio frequency dataset and a handcrafted signal sampling dataset and discusses the experimental results. Finally, Section 6 concludes the paper.

## 2. Related Works

AMR is a typical multiclass classification problem in the communication domain. This section introduces several deep learning methods for AMR and presents some of the latest developments in deep learning that we want to introduce to the AMR field.

### 2.1. Deep Learning for AMR

Thanks to the advancement of parallel computing power and the development of big data, deep learning has recently achieved great success. Wireless communication engineers have been adapting the latest deep learning technology to signal recognition. Several works have been focusing on applying DL to AMR scenarios. Azzous et al. [11], who were pioneers in this field, proposed using an artificial neural network (ANN) to classify analog modulated communication signals. This attempt inspired the idea of applying deep learning to AMR. O’Shea et al. [12,13,14] proposed a method for applying a Convolutional Neural Network (CNN) to the field of modulation recognition, by using the time domain in-phase and quadrature (IQ) signal as the input to the network. An even more valuable contribution is that they published a dataset containing multiple standard modulations that has subsequently become a standard for the AMR domain. Khan et al. [15] proposed employing signals of asynchronous delay tap plots (ADTPs) to train deep neural networks (DNNs) for joint modulation format/bit-rate classification, and verified the stability of their method under multipath channel simulations. Li et al. [16] proposed an algorithm based on anti-noise processing and a deep sparse-filtering convolutional neural network (CNN). This method uses the CNN to extract the signal cycle spectrum, which significantly increases the robustness of the algorithm under low signal-to-noise ratio (SNR) conditions. This method was essentially based on manual feature extraction and adaptive classification. Bin et al. [17] successfully linked signal processing with computer vision by application of GAN. The core idea is to extract constellation diagram as features for modulation classification. Zhang et al. [18] proposed a fusion model to process signal data directly. The model includes both a CNN and a long short-term memory (LSTM) [19]. This method is an integrated learning approach. Hauser et al. [20] discussed how the classification performance of modulation signals is affected by sampling rate offsets and frequency offsets. This research demonstrates that training CNN over frequency and sample rate offsets does not have significant impact on performance.

We believe that the existing methods have two main characteristics: they consider only supervised learning, and most existing methods involve signal preprocessing and do not fully exploit the feature-extraction capabilities of deep learning:

(1) For the first characteristic, most of the existing independent studies analyzed the supervised learning scenario. These studies explored the impacts of network structure and signal representation on classification results. They have achieved good results in this scenario. However, most of these efforts were conducted with ideal amounts of training data. They did not explore the use of AMR methods when missing labeled training data. These problems are more likely to occur when dealing with unknown tasks and signals, such as signal acquisition in non-cooperative situations. Additionally, Tang Bin et al. [17] applies GAN as an approach of data augmentation. But in essence, it is based on sufficient amount of labeled data.
(2) Regarding the second characteristic, most of the existing methods are carried out by extracting features firstly and then performing classification. This approach is not fundamentally different from traditional expert-knowledge-based modulation recognition, except that the deep neural network is regarded as an enhanced classification tool. In the realization of cognitive radio, this limited the ability of systems to adapt to unknown and new tasks. We believe that this solution does not fully exploit the capabilities of deep neural networks for extracting features—it is simply a compromise to offset the complexity of training deep learning models. In contrast, our method uses the IQ signal as input and relies on appropriate network design to achieve better classification results. This is a major difference between our approach and the existing preprocessing methods.

### 2.2. Generative Adversarial Networks

Before GANs were proposed, conducting unsupervised learning was a difficult problem, whether the target was a classic probability map-based model or a CNN (although CNNs are surprisingly effective in supervised learning tasks such as classification and segmentation). Goodfellow et al. [10] improved the situation by proposing GANs. GAN architecture includes two networks: a generator (G) and a discriminator (D), both of which are common convolutional. The former generates a sample from a random vector, and the latter discriminates between the generated sample and the training set samples. These two networks are trained simultaneously. The training goals are to minimize the discriminant error when training the discriminator and maximize the discriminant error when training the generator. Both goals can be achieved using a backward propagation method. A well-trained generative network can transform noise vector into a sample similar to those in the training set. This noise involves the encoding of the sample in a low dimensional space. At the same time, the trained discriminator can discriminate between real and fake samples.

The raw GAN method does not require pre-modeling, but unstable training usually occurs with larger images or pixels. To solve this problem, Mirza et al. [21] proposed the conditional generative adversarial network (CGAN). By adding constraints to GANs, that is, adding conditional variables during the modeling of D and G, they were able to achieve more stable training. Later, Radford et al. [22] proposed a model called DCGAN, revealing many architectural designs that are important for offsetting GAN’s unstable learning style and gaining specific training examples for CNN networks. DCGAN introduced the Batch-Normalize (BN) [23] transform to the G and D networks to avoid collapse. Larsen et al. [24] proposed a combination of variational autoencoder (VAE) and GANs to reuse the features developed by GANs in VAE’s reconstruction objective, which combined the advantages of GANs and VAE.

### 2.3. Spatial Transformer Networks

CNN achieves translation invariance by introducing max-pooling layers. However, due to the typically small spatial variability for max-pooling, CNN lacks feature invariance for some artificially transformations such as rotation, distortion, etc. In CR, the raw IQ two-way signal can only be pooled in time domain, so the no-morphing of the CNN pooling is limited. If the input image is subjected to a wide range of translation transformations, the feature map still cannot be invariant. Jaderberg et al. [25] proposed the Spatial Transformer Network (STN), which generates a corresponding parameter of the spatial transform for any input image or feature map. Then, according to this parameter, the original image is subjected to a global spatial transformation to obtain the final standard pose. The model consists of a trained localization network that performs a regression parametric transform operation and a trained discriminative classifier that selects a class estimate. The spatial transformation parameter of the input image can be learned in a data-driven manner, and the network becomes spatially invariant. We introduce this data standard method into the field of modulation identification having attention model as basis. STN is the theoretical basis of our design of signal synchronization and normalization module (SSTM). 

## 3. Generative Adversarial Network Design

The model we are proposing here is a data-driven end-to-end framework. Collecting sufficient training data is the basis for an effective machine learning model. Therefore, before performing deep neural network design, we first define the representation of wireless signals.

The data acquisition process of the wireless signal includes: amplifying, mixing, low-pass filtering, and analog-to-digital conversion after obtaining the received signal r(t). We perform *n*th point sampling on the IQ signal. The values are expressed as r[n], including two components, the quadrature component, $r_{Q}[n]$ and the in-phase components, $r_{I}[n]$. These two components are orthogonal to each other. Then there is an array of discrete wireless signal samples with length *n* r[n], n=0,…,n−1. A vector is used to represent the signal, that is:

(1) $$r_{raw}={\left[r[0],r[1],\ldots r[N-1]\right]}^{T}\;$$

This vector is a segmented representation of the signal, containing the time stability characteristics of the signal. We express the signal by IQ in two ways:

(2) $$r_{raw}={\left[r_{I}[0],r_{I}[1],\ldots r_{I}[N-1]\right]}^{T}+j{\left[r_{Q}[0],r_{Q}[1],\ldots r_{Q}[N-1]\right]}^{T}\;$$

The orthogonal two-way signal is represented as follows:

(3) $$\begin{array}{l} r_{I}=[r_{I}[0],r_{I}[1],\ldots r_{I}[n-1]]T\\ r_{j}=[r_{Q}[0],r_{Q}[1],\ldots r_{Q}[n-1]]T \end{array}$$

Then the raw signal can be expressed as follows:

(4) $$r_{raw}=r_{I}+jr_{Q}\;$$

Then the IQ vector samples vector r is mapped into two sets of real-valued data vectors, that is:

(5) $$r={\left[\begin{array}{c} r_{I}\\ r_{Q} \end{array}\right]}^{T}\;$$

so that the data vector $r\in C^{N}$ is translated into $r\in R^{2\times N}$, this can be expressed mathematically as:

(6) $$f:\;\begin{array}{c} C^{N}\to R^{2\times N}\\ r_{raw}\to r \end{array}\;$$

We represent the signal as a $R^{2\times N}$ vector. So the input to the end-to-end system has been determined. The system output l is defined as the output of the signal sample categories. Dataset *S* is then obtained as *m* input-output pairs, can be denoted by:

(7) $$S=\left\{\left(r_{1},l_{2}\right),\left(r_{2},l_{2}\right),\ldots ,\left(r_{m},l_{m}\right)\right\}\;$$

### 3.1. Framework

In this section, a new signal modulation deep learning method based on the idea of generative adversarial network is proposed. This method is appropriate for semi-supervised learning. In the case of insufficient data, due to the contradiction between generalization and fitting of deep learning, it is often difficult to achieve good results in actual samples testing. Therefore, we use GANs to learn essential features of signal modulation and maximize the effect of capacity advantage of deep neural networks.

The core of training GAN is reaching the Nash equilibrium point. Nash equilibrium, also known as non-cooperative game equilibrium, is an important strategy of the game theory. GAN tries to reach the Nash equilibrium point through the confrontation training. At the Nash equilibrium point, both networks reach their optimal performances from the perspective of the game theory. In the training process, we find that the classical GANs and the CGAN with conditional constraints are difficult to converge [26]. This is due to the training instability of GAN, specifically manifest in training process as the occurrence of mode collapse. This issue is discussed in this section. 

When mode collapse occurs, the generation diversity is insufficient. It is difficult for generator to fit the complex modulation signal distribution. The distribution of the real samples is represented by $P_{data}$, and the distribution of the generated samples is represented by $P_{G}$. The optimization goal of the generator is to approximate the distribution of the real samples and the fake samples. The similarity of the two distributions is described by the Kullback–Leibler (*KL*) divergence:

(8) $$KL=\int P_{data}\log\frac{P_{data}}{P_{G}}dx\;$$

The goal of the generator is to minimize the *KL* divergence, acknowledged as relative entropy. For the classical *KL* divergence, there is an infinite *KL* divergence because $P_{data}$ has a value and $P_{G}$ has no value. When we have a value of $P_{data}$, we guarantee that $P_{G}$ also has value, which can ensure that *KL* divergence does not tend to infinity. If our G capacity is not enough, we can only produce some simple Gaussian-like distributions. So G tends to generate more secure samples. Although there is basically no situation of mode collapse at this time, there will be many meaningless samples, and it is difficult to achieve the Nash equilibrium of G and D. For improved reverse *KL* divergence:

(9) $$reverseKL=\int P_{G}\log\frac{P_{G}}{P_{data}}dx\;$$

If there is a $P_{G}$ that produces a value in a position where there is no $P_{data}$ ($p_{data}\approx 0$), it will make this reverse *KL* divergence positive infinity value. Therefore, for the training process of minimizing *KL* divergence, there will be a high penalty. For safety, $P_{G}$ will be more inclined to generate the homologous stability samples that will be considered as real, and will not risk generating some different samples. So the generator will prone to completely fit some data distributions which are easy to learn, then mode collapse occurs [27]. There are some issues that need to be addressed when training GAN in AMR applications:

- Due to the complexity of the modulation signals, mapping the high-dimensional parameter space to a low-dimensional classification space is prone to get trapped in non-convergence.
- The traditional GAN model is easy to mode collapse, sine the lack of generated samples’ diversity.

**Figure 1:**
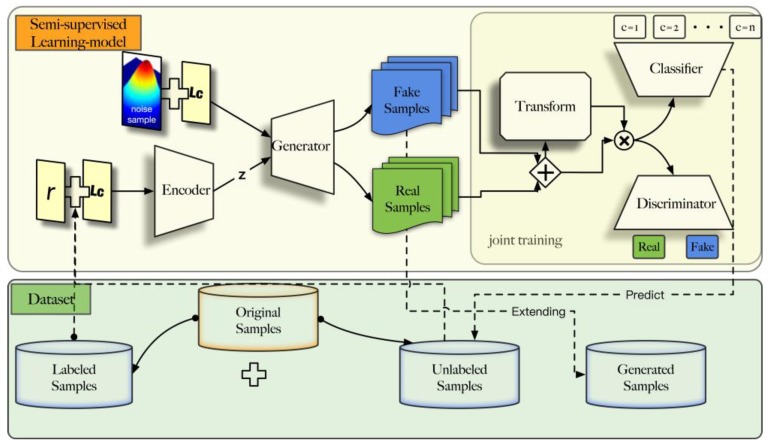
Framework of the generative adversarial networks (GANs)-based semi-supervised learning method.

To solve these problems, we used the framework of Conditional Generative Adversarial Network (CGAN) [21] and Auxiliary Classifier Generative Adversarial Network (ACGAN) [28]. CGAN and ACGAN add category information to the input of the generator. By adding this a priori information, it can better guide the data enhancement of the generator. In D, we not only require the output of the true and fake information of the sample, but also the output of the category information. However, in the course of the experiment, it was still difficult to obtain satisfactory results through this framework. The network structure needs to be further optimized. Inspired by the conditional variational auto-encoder (CVAE) and the spatial transformer network (STN), we insert an encoder prior to the generator that simultaneously splits the classification and discriminating functions of D. Besides, we use two independent networks to handle classification and discriminating functions. Each of the two networks optimizes through backward propagation. An attention module named SSTM based on STN is proposed to target wireless signals. This improved framework is shown in Figure 1.

As shown in Figure 1, the proposed method consists of five parts: encoder network (E), generator network (G), signal spatial transform module (T), classifier network (C), and discriminator network (D). r represents a signal sample defined in Equation (7), and $L_{c}$ represents the category label of r. E learns the data distribution P(z | r,c) to map the data sample r to the hidden variable representation P(z | r,c). G in our framework learns the distribution P(r|z,c) through the hidden variable generated by E to generate real samples. For a real sample, it generates hidden variable by passing E. Then, the real sample is generated by decoding of the hidden variable through G. For a fake sample, it is generated by decoding with random vector. This combination of E and G is inspired by CVAE. We split the function of the discriminator network in ACGAN and assigned the two tasks of classification and discrimination to two independent networks. The discriminator network D has the same function as the classic GANs. The classification network C focuses on the posterior P(c | x). To adapt to the AMR task, inspired by the STN, a Signal Spatial Transformer Module (SSTM) is added before the classify and discriminate process. This module functions as the network T. to solve the instability of GAN training. When SSTM is added to the network, the signal samples are normalized before entering the subnetworks. The normalized process reduces the complexity of data distribution of signal samples, simplifies the tasks of subnetworks.

In this section, the optimization goals for each part of the network is defined. Assume we have a labeled dataset S in which the labels belong to K categories. During the sample generation process, we use E to establish the mapping between real space $R^{2\times N}$ and latent space z. This method clearly establishes the relationship between the generated sample and the real sample, which reduces the difficulty of training G. In addition, this method effectively reduces the mode collapse problem. VAE is a classic generation model, which consists of a coding network and a decoding network, which can be regarded as the processes of information compression and decompression. VAE uses E to generate a z as close as possible to the specified distribution. However, by sampling z from this distribution, valid data can be obtained through G. Its optimization goal is to make the raw data sample and the generated data as similar as possible at the pixel levels. This method often produces blurred images. Like VAE, E processes the covariance ε and mean μ of the latent vector. The *KL* loss is calculated to evaluate the disparity between the proposed distributions and the prior P(z), i.e.,:

(10) $$L_{KL}=\frac{1}{2}\left(\mu^{T}\mu +sum\left(e^{\varepsilon }-\varepsilon -1\right)\right)\;$$

Then the latent variable *z* is sampled according to the mode z=μ+s⊙exp(ε), where s∈N(0,I) is a random matrix that satisfies the normal distribution. After acquiring the latent variable z, we generate fake samples through G. This approach constitutes an improvement of the generative adversarial network, that is, the loss of D and C is added to the loss of the generator. Here, we define $D_{f}$ as the feature map of the last fully connected layer output, and define $C_{f}$ as the feature map of the last fully connected layer output of C. Thus, the optimization goal of the generator is calculated as follows:

(11) $$L_{G}=\frac{1}{2}({\left\|r-r^{\prime }\right\|}_{2}^{2}+{\left\|D_{f}(r)-D_{f}(G(z))\right\|}_{2}^{2}+{\left\|C_{f}(r)-C_{f}(G(z))\right\|}_{2}^{2})\;$$

where *D*(*r*) represents the D’s prediction on real samples and *G*(*z*) represents generated samples from G. *D*(*G*(*z*)) represents the D’s prediction on fake samples. D’s optimization goal of D is defined as:

(12) $$L_{D}=-E_{r\sim P_{data}}[\log D\left(r\right)]-E_{z\sim P_{z}}[\log\left(1-D\left(z\right)\right)]\;$$

The feedback optimization given by G for the loss of D is defined as $L_{GD}$. We define $D_{f}$ as the feature map of the last fully connected output layer. By using the loss function of feature map matching, the loss is calculated as follows:

(13) $$L_{GD}=\frac{1}{2}{\left\|E_{r\sim P_{data}}D_{f}\left(r\right)-E_{z\sim P_{z}}D_{f}\left(G\left(z\right)\right)\right\|}_{2}^{2}\;$$

In the classifier design, we use the standard softmax as the classification loss. The discriminator outputs a *k* dimensional vector of logical values $C=\left\{l_{1},l_{2}\ldots l_{k}\right\}$. The vector is transformed into $P=\left\{P_{1},P_{2},P_{k}\right\}$ by softmax function, where:

(14) $$P_{j}=\frac{e^{lj}}{\sum_{i=1}^{k+1}e^{l2}}j\in \left\{1,2,\ldots ,k\right\}\;$$

The combination of the $P_{j}$ values represents the posterior probability P(c|r), then, the network tries to minimize the classification loss:

(15) $$L_{c}=-E_{r∼p_{data}}\left[\log p\left(c|r\right)\right]\;$$

Feature map matching is used for G’s feedback to C. Here, we define $C_{f}$ as the feature map of the last fully connected layer output of C. Then the loss function of G feedback on C is defined as follows:

(16) $$L_{GC}=\frac{1}{2}\sum_{C}\left\|E_{r∼P_{data}∼}C_{f}\left(x\right)-E_{z∼P_{z}}C_{f}\left(G\left(z,c\right)\right)\right\|\;$$

Thus, the min-max game loss of the entire network can be defined as:

(17) $$L=L_{D}+L_{C}+\lambda_{1}L_{KL}+\lambda_{2}L_{GD}+\lambda_{3}L_{GC}+\lambda_{4}L_{G}\;$$

where V(G,D,C) is the combined loss. For G, the optimization problem is:

(18) $$\begin{array}{ll} \underset{G}{\min}V\left(G,D,C\right)= & \underset{G}{\min}(\frac{\lambda_{1}}{2}{\left\|E_{r∼P_{data}}D_{f}\left(r\right)-E_{r\sim \;pz}D_{f}\left(G\left(z,c\right)\right)\right\|}_{2}^{2}\\ & \;+\frac{\lambda_{2}}{2}{\left\|E_{r\sim \;P_{data}}C_{f}\left(r\right)-E_{r∼P_{z}}C_{f}\left(G\left(z,c\right)\right)\right\|}_{2}^{2}\;\\ & \;+\frac{\lambda_{3}}{2}({\left\|r-r^{\prime }\right\|}_{2}^{2}+{\left\|D_{f}\left(r\prime \right)\right\|}_{2}^{2}+{\left\|C_{f}\left(r\right)-C_{f}\left(r\prime \right)\right\|}_{2}^{2}))\\ & \;=\underset{G}{\min}\left(\lambda_{2}L_{G}+\lambda_{3}L_{GD}+\lambda_{4}L_{GC}\right) \end{array}$$

Thus, the goal of D and C can be defined as follows:

(19) $$\underset{D}{\max}V\left(D,G,C\right)=\underset{D}{\max}\left[E_{r∼p_{data}}\left(\log\left(r\right)\right)+E_{z∼p_{z}}\left(\log\left(1-D\left(G\left(z\right)\right)\right)\right)\right]\;$$

(20) $$\underset{C}{\max}V\left(D,G,C\right)=\underset{C}{\max}\left[E_{r∼p_{data}}\left(\log P\left(c|r\right)\right)\right]\;$$

All of these loss functions in Equations (10)–(20) complement each other. $L_{KL}$ is only relevant to E. It indicates whether the distribution of latent variables is within expectations. $L_{C}$ is related to the C network, which represents the ability of the network to classify samples from different categories. $L_{G},\;L_{GC},\;L_{GD}$ are relevant to G, which indicate the similarity between generated samples and real samples, training samples and same category samples. $L_{D}$ is relevant to D. It represents the capability of D to distinguish fake samples. The application of all these loss functions is described in the algorithm of Section 4.3 Implementation Details.

### 3.2. Signal Spatial Transformer Module

STN is a method for adaptively deforming images in deep learning. It can solve the problem that the convolution network lacks the ability to spatially transform data. The STN module consists of a subnetwork and affine transformation functions that predict the transform parameters. The classic affine transformation is expressed as follows:

(21) $$\left[\begin{array}{c} \theta_{11}\\ \theta_{21} \end{array}\begin{array}{c} \theta_{12}\\ \theta_{22} \end{array}\begin{array}{c} \theta_{13}\\ \theta_{23} \end{array}\right]\left[\begin{array}{c} x^{source}\\ y^{source}\\ 1 \end{array}\right]=\left[\begin{array}{c} x^{T\arg et}\\ y^{T\arg et} \end{array}\right]\;$$

where, $\left(x^{Source},y^{Source}\right)\left(x^{\mathrm{Target}},y^{\mathrm{Target}}\right)$ represents the original image pixel point, θ represents the affine transformed pixel point, and the coefficient matrix is the affine transformation coefficient, which can be used to adjust the coefficient matrix to achieve image enlargement, reduction, translation, rotation, etc.

The STN network does not need to calibrate key points and can adaptively convert and align the data spatially. When the input data have large spatial differences, the generalization performance of the network can be improved by the STN. We introduce the idea of adaptive affine transformation into the AMR domain to normalize the signal. Considering the signal distortion caused by the channel effect and errors caused by the transmitter and receiver hardware during signal transmission, the receiver will output a damaged signal. The most common causes of signal distortion are as follows:

- Frequency Offset: Frequency offset is caused by differences in the local oscillator frequency between the receiver $f_{c}$ and the transmitter ${f_{c}}^{\prime }$
- Phase offset: φ(t) time drift is caused by the frequency offset of the local oscillator (LO).
- Timing drift: Timing drift is caused by different sampling rates.
- Noise: Noise introduced by components such as antennas, receivers, etc. $n∼N\left(0,\sigma^{2}\right)$ is used to model this interference.

Therefore, the signal output by the receiver can be expressed as:

(22) $$r\left(t\right)=\left(s\left(t\right)*h\left(t-\tau \right)\right)\cdot e^{j2\pi \left(f_{c}-f_{c}^{\prime }\right)+\varphi (t)}+n\left(t\right)\;$$

In the above formula, r(t) is the data output by the receiver, s(t) is the bandpass signal with a center frequency of $f_{c}$, n(t) is the noise contribution and h(t−τ) is the channel response. 

**Figure 2:**
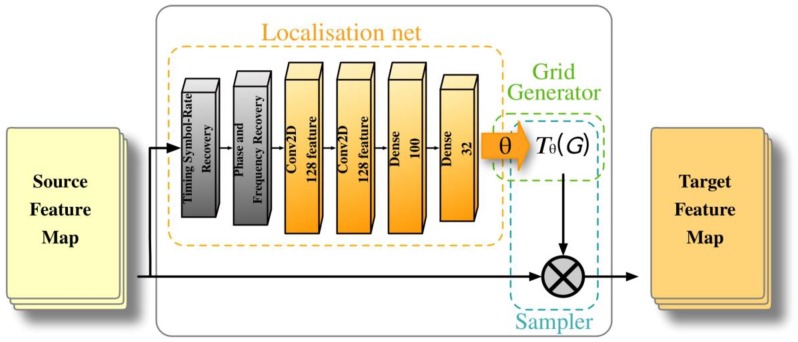
Structure of SSTM.

SSTM corrects signal distortion caused by frequency offset, phase offset and time drift through parameter learning. Figure 2 shows the structure of our proposed SSTM. We consider time drift as sampling with the correct offset, which is similar to the attention mechanism for images, therefore, we directly use the affine transform in the image domain for processing. A 2 × 6 parameter vector is used to process the 2D affine transformation. This parameter vector is defined as follows:

(23) $$\left[\begin{array}{c} \theta_{11}\\ \theta_{21} \end{array}\begin{array}{c} \theta_{12}\\ \theta_{22} \end{array}\begin{array}{c} \theta_{13}\\ \theta_{23} \end{array}\right]\;$$

We simulate frequency and phase offset directly by signal transformation and register them as one of the localization nets using:

(24) $$y_{n}=x_{n}e^{n\theta_{3}+\theta_{4}}\;$$

where $\theta_{3}$, $\theta_{4}$ represents $f_{c}-f_{c}^{\prime }$, φ(t) in Equation (22).

## 4. Evaluation Setup

In this section, a framework for the radio modulation signal classification is constructed. Besides, we define the dataset used for training and evaluation, the structure of each subnetwork, other implementation details, and the scheme for evaluating the classification performance. These details are necessary for implementation of the experiment.

### 4.1. Dataset Description

To evaluate end-to-end wireless modulation recognition system framework, we used synthetic RF data set [29] generated by researchers at Virginia Tech. The GNU Radio dataset is a basis for evaluating the modulation recognition task. The data set contains signals of 11 modulation modes, AM-DSB, AM-SSB, 8-PSK, 4-PAM, 16-QAM, WBFM, 64-QAM, GFSK, QPSK, BPSK and CPFSK. The dataset includes 1100 training examples and 1100 testing examples for each modulation type. The training set and testing set are spliced by random extraction. Each sample contains 8–16 symbols. The samples were generated at various SNRs, ranging from −20 dB–20 dB. The signals are subjected to channel distortion, frequency offset, phase offset, and Gaussian noise as described in the aforementioned channel model. The shape of each data sample is $r\in R^{2\times 128}$, and the data label is divided into an authenticity label $l_{s}\in R^{2}$ and a category label $l_{C}\in R^{11}$.

### 4.2. Network Structure

The structure of each subnetwork is individually designed. For subnetworks C and D, which are responsible for signal feature extraction, we use a standard CNN network architecture. The input tensor for the first layer is 2 × 128, and it receives r as the input data. The network uses typical CNN convolutional-plus-pooling layers to reduce the feature map. The C network outputs a one-hot encoded category, $l_{C}\in R^{11}$, and the D network outputs the authenticity as a one-hot encoded truth value, $l_{s}\in \sim^{2}$. An important principle we observe in designing networks structures is that D and G should have opposite structures. This is proposed by Radford, et al. [21], and this improvement significantly reduces the uncoordinated updating between layers, which helps to stabilize training. In addition, the functions of both D and C are to obtain a low-dimensional representation of the signal. Therefore, we derive the network structures of D, G and E by separately designing C network. The structures of C and D are shown in Table 1.

The E subnetwork attempts to extract the original sampling features. After the original input passes the encoder, E extracts the mean and log variance. These two values are random numbers of different features for the samples with different features. Then, the two values are used subsequently. The characteristics (mean and variance) of the random number are generated so that E will retain a correlation with the input. To extract the temporal correlation characteristics of the data and avoid repeatedly extracting features with the C and D networks, we use the feature extractor of the long short-term memory (LSTM) structure in E’s structure. E converts the input $r\in R^{2\times 128}$ into two m-dimensional values: the first is the mean of 100 Gaussian distributions, and the second is the logarithm of 100 Gaussian distribution variances, generated according to the above two m-dimensional data and M-dimensional random numbers obeying a Gaussian distribution. The structure of E is shown in Table 2.

G network generates fake samples from the latent variables and the predicted category labels. We use a structure similar to DCGAN, which simplifies training. G has a structure, similar to C but performs a reverse generation, including deconvolutional and up-sampling operations. The structure of G is shown in Table 3.

For the T network, we extract the affine transformation parameters through a specially designed SSTM module. A classic CNN network with the phase offset and frequency offset extraction layer is applied. Various connection methods, layer depth, kernel numbers are evaluated in Section 4.3.

### 4.3. Implementation Details

**Figure 3:**
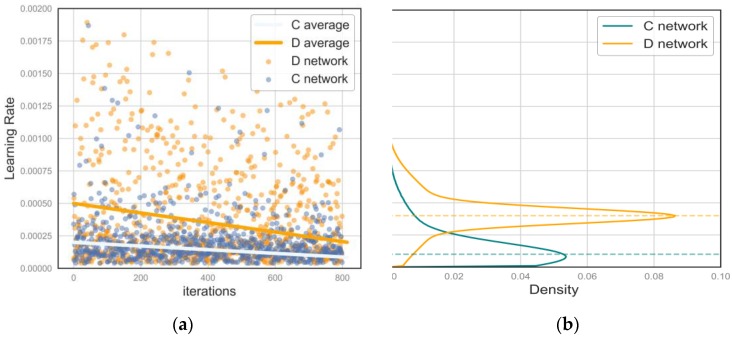
Optimal hyperparameters establishment: (**a**) Search processing of optimal hyperparameters with iterations and hyperparameters fitting using a sliding average; (**b**) Density map of the global hyperparameters.

Keras [30] and tensorflow [31] were used for mixed programming. We use keras definitions for standard layers such as convolution, deconvolution and pooling. We also mixed tensorflow to define custom layers in SSTM and mini-batch layers. The employed deep learning workstation was equipped with an NVIDIA 1080Ti graphics card and high-performance central processing unit (CPU) with 16 GB of random access memory (RAM). Deep learning algorithms are highly dependent on the choice of hyper parameters. We attempt to find the optimal learning rate through Bayesian optimization. For GANs, the loss of each network converges during the min-max game, and it is difficult to evaluate the impact of the choice of learning rate on performance. However, according to prior research, G and D should use the same learning rate to reduce the chance of crashes and mode collapse according to Goodfellow et al. [32]. We separately train D and G to find the optimal learning rate. We use the open source Hyperopt [33] library to perform Bayesian optimization [34] on the C and D network. Parameter tuning, and the optimal learning rate were estimated based on the kernel density, as shown in Figure 3. The dotted line in Figure 3 represents the optimal learning rate we obtained.

G uses the RMSPROP optimizer, C and D use the typical ADAM [35] optimizer. To maintain training stability, some tricks mentioned in the literature [26] are used, including the latest technologies to prevent overfitting and accelerate training. One of them is label smoothing. We smooth the labels $l_{s}$ and $l_{C}$ by adding noise to the value of the labels. Another trick is batch normalization. We apply batch normalization by ensuring that there are only real samples or fake samples in a mini batch. Using a mix of true and fake samples in a mini batch can result in gradient instability. The hyperparameters defined in this work are shown in Table 4.

**Figure 4:**
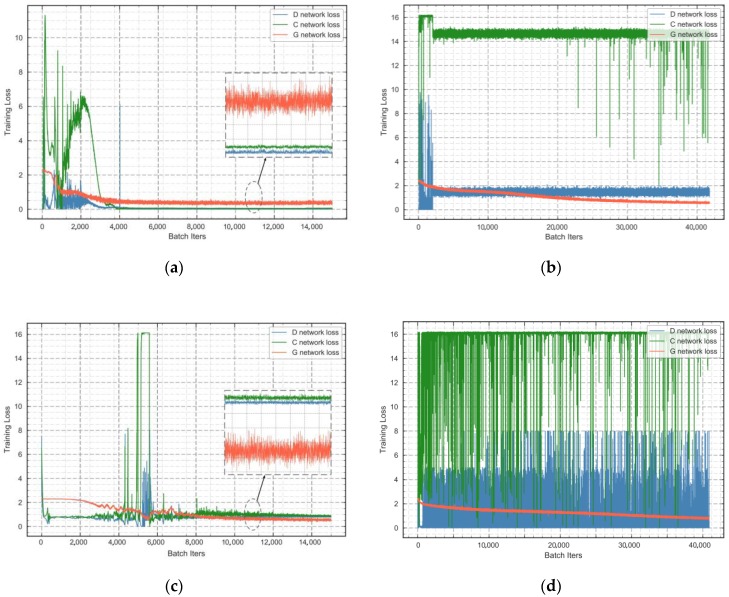
Loss convergence with regard to training epochs for various frameworks: (**a**) SCGAN; (**b**) SCGAN without SSTM; (**c**) ACGAN with SSTM; (**d**) ACGAN without SSTM.

In our framework, the introduction of SSTM improves training stability and ultimately leads to performance improvement. We compare the loss functions of several frameworks with or without SSTM in the Figure 4. Experiments are taken on ACGAN with SSTM, ACGAN, SCGAN with SSTM and SCGAN without SSTM. For these frameworks, we compare training time and convergence by analyzing loss function of subnetworks. As presented in Figure 4, the comparison between SCGAN with SSTM and other none SSTM frameworks indicates the advantage of SSTM. Figure 4a shows that SCGAN with SSTM converges on 4000 batch iterations. The loss of C is 0.45 at this point. But as shown in Figure 4c, ACGAN with SSTM converge on 9000 batch iterations with loss function of 1.2. Compared with ACGAN with SSTM, SCGAN achieves convergence faster. At the same time, the classification loss is smaller. In Figure 4b,d, none SSTM frameworks get mode collapse. This result shows that SSTM can improve training stability and convergence speed. The improvement of training stability is the premise of our application of GAN framework.

**Figure 5:**
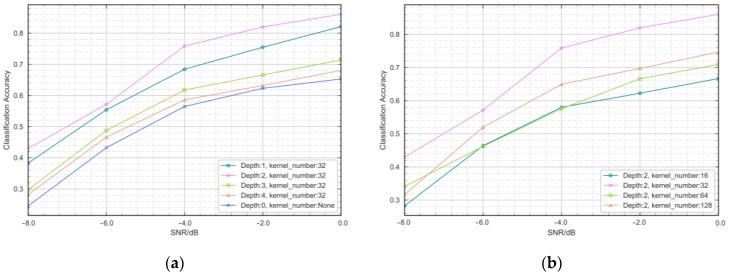
Correct recognition percentages versus SSTM structure.

We conducted a comprehensive study to select the network structure that is most appropriate for AMR tasks. We analyzed the impact of SSTM network depth and the number of convolutional feature maps on model performance. The effect of layer depth and number of feature maps on accuracy is shown in Figure 5. It can be seen that for model depth, we compared the effects of no SSTM and the accuracy of the SSTM using one to four convolutional layers. Preliminary analysis shows that training stability is improved after using the SSTM. When the depth of the model is more than two convolutional layers, increasing the depth does not bring about an improvement in performance. This issue has been verified in [14]. Because of the similarity of subnetwork structure and CNN, we believe that this situation is in line with expectations. In addition, as shown in Figure 5b, it can be seen that when the kernel number is 32, the model performance is outstanding, and more feature maps will cause a visible performance decline. We have two surmises about this. One is that the increase in complexity leads to performance decline. Second, the redundant information brought by more feature maps leads to the instability of adversarial training. 

The specific training process is shown in Algorithm 1. For the labeled data, E, G, C and D are updated. The core idea of semi-supervised learning with SCGAN is as followed: When using traditional GAN, the unlabeled sample does not have a category label, but it has the artificial attribute of real, so this information can be used to train G and D. In SCGAN, this idea is also applies, unlabeled samples are used to update E, D and G. The training process is divided into the following steps. First, the unlabeled samples forwards C to obtain the predicted category $l_{p}$. Then, the calculation and gradient descent of each network are performed separately, but the backward propagation is not performed on C. After this process, SCGAN is updated in semi-supervised way.

**Algorithm 1.** Semi-Supervised Learning through Asymmetric Training**Input:** Original training data set *S*, number of total epoch iterations *I*, batch size *N*, *C* network parameters, $\lambda_{1}$, $\lambda_{2}$, $\lambda_{3}$, $\lambda_{4}$ **Output:** Vectors of class probabilities *h1*
**1.** 
**Initialization**
**2.** **Joint training:** repeat until *I* epoch. **for** *i* = 1 **to** *I* **do**         Dram samples $\left\{r_{m},l_{m}\right\}\sim P_{data}$ from data set *S*.         **While** $\left\{r_{m},l_{m}\right\}\sim P_{data}$ **is** labeled samples               Calculate $L_{KL}$ with $KL\left(q(z|r_{m},l_{m})\|P_{z}\right)$.               Generate z with $E\left(r_{m},l_{m}\right)$.               Generate encoder samples $r_{e}$ with $G\left(z,l_{m}\right)$.               Draw noise samples $z_{f}\sim P_{z}$ from noise prior, sample $l_{f}$ of random class.               Generate $r_{f}$ with $G\left(z_{f},l_{f}\right)$.               Calculate loss function of D $L_{D}$.               Obtain the feature center $\frac{1}{N}\sum_{i}^{N}D_{f}\left(r_{m}\right)$ of $r_{e}$, and $\frac{1}{N}\sum_{i}^{N}D_{f}\left(r_{f}\right)$ of $r_{f}$.               Calculate loss function of D on G $L_{GD}$.               Calculate feature center $C_{f}^{l_{i}}\left(r_{m}\right)$ of each class $l_{i}$ for $r_{m}$, and $C_{f}^{l_{i}}\left(r_{f}\right)$ for $r_{f}$.               Calculate loss function $L_{GC}$ of C on G.               Calculate G loss $L_{G}$.               Perform gradient descent on the parameters of D with $L_{D}$.               Perform gradient descent on the parameters of C with $L_{C}$.               Perform gradient descent on the parameters of G with $\lambda_{2}L_{G}+\lambda_{3}L_{GD}+\lambda_{4}L_{GC}$.               Perform gradient descent on the parameters of E with $\lambda_{1}L_{KL}+\lambda_{2}L_{G}$.         **Else** $\left\{r_{m}\right\}\sim P_{data}$ **is** unlabeled samples               Predict label with $l_{p}\sim C\left(r_{\varepsilon }\right)$.               Calculate $L_{KL}$ with $KL\left(q(z|r_{m},l_{p})\|P_{z}\right)$.               Generate z with $E\left(r_{m},l_{p}\right)$.               Generate encoder samples $r_{e}$ with $G\left(z,l_{p}\right)$.               Draw noise samples $z_{f}\sim P_{z}$ from the noise prior, sample $l_{f}$ of the random class.               Generate $r_{f}$ with $G\left(z_{f},l_{f}\right)$.               Perform gradient descent on the parameters of D with $L_{D}$.               Perform gradient descent on the parameters of G with $\lambda_{2}L_{G}+\lambda_{3}L_{GD}+\lambda_{4}L_{GC}$.               Perform gradient descent on the parameters of E with $\lambda_{1}L_{KL}+\lambda_{2}L_{G}$.
**end for**
**3.** 
**Output:** *h1.*



## 5. Numerical Results

The results analysis consists of three parts. Firstly, the effectiveness of our proposed method under the supervision conditions and evaluates the classification effect on the RadioML [29] data set are verified. Then, we prove its performance under semi-supervised conditions and compare it with the classical semi-supervised learning method. Besides, we perform a systematic verification of the generated fake samples. The numerical characteristics of the fake samples are compared with those of the real samples. This method can be used both to evaluate the performance of the generator and to address possible signal-level phishing attacks in the future.

### 5.1. Classification Performance

The SCGAN is trained on the training data set to evaluate the performance of our method and the network reaches convergence (Nash equilibrium) after approximately 300 epochs. Then, we obtained effective generation and the discriminant models (C and D). We use D to test on the verification set. The average correct classification rate (CCR) is calculated on all methods. We compared our SCGAN with other five methods: the support vector machine (SVM), naïve Bayes (NB), CNN and ACGAN.

**Figure 6:**
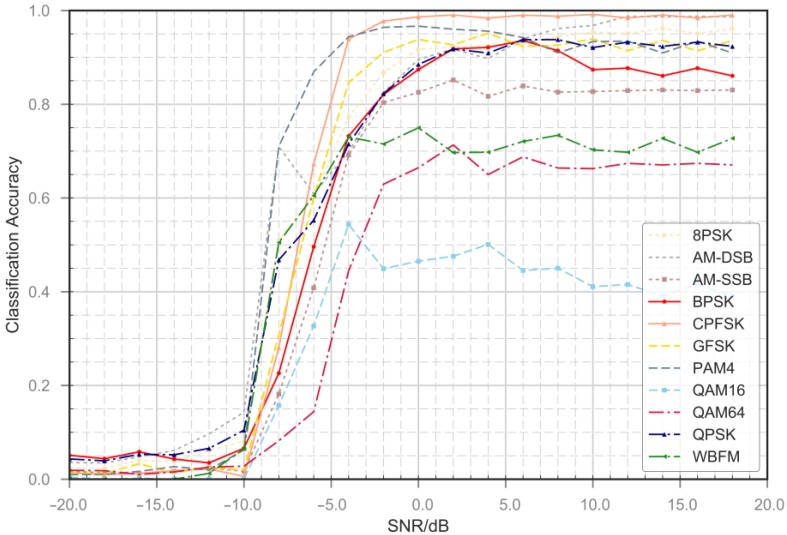
Correct recognition percentages versus SNR for all types of modulation signals.

**Figure 7:**
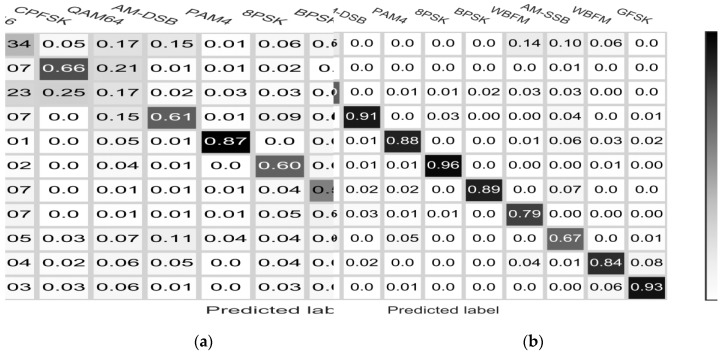
Confusion matrices for the modulation recognition data in SNR: (**a**) 0 dB; (**b**) 5 dB.

**Figure 8:**
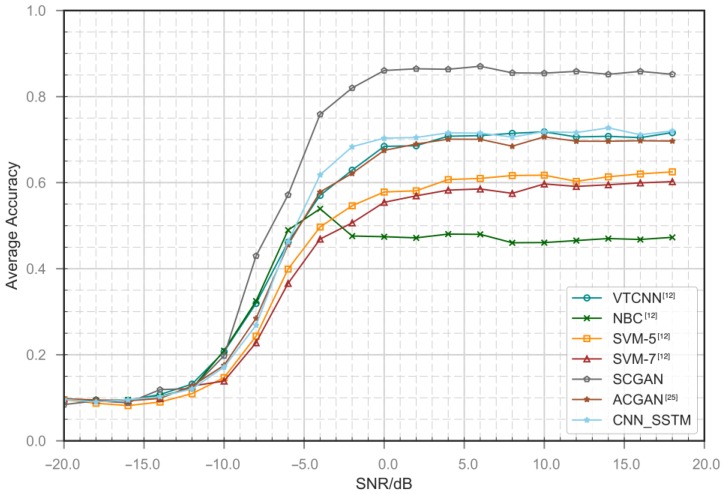
Overall classification accuracy versus SNR.

Our preliminary results shown in Figure 6 and Figure 7, indicate that our method performs well at medium and high SNR s on all types of modulation signals. The classification accuracy increases as the SNR increases. For AM-DSB, 8PSK, BPSK, CPFSK and other signal types, the classifier shows good performance. When the signal-to-noise ratio rises from −10 dB to 0 dB, the classification accuracy rises sharply. As shown in Figure 8, we compare the average accuracy of our methods; our method performs extraordinarily better than the classical machine learning methods and the simple CNN method. In the legend of Figure 8, SVM-5 and SVM-7 represent integrated classifier consisting of five and seven SVM classifier. Naïve Bayes classifier (NBC) and VTCNN represent the naïve Bayes classifier and CNN classification network proposed by O’Shea [12]. In addition, we add SSTM to the VTCNN method and compare its performance gains. SSTM is joined before VTCNN. We also test the ACGAN method. The subnetworks of ACGAN share the same structures with our framework, but we do not use the additional structure to avoid mode collapse, it is difficult to achieve convergence. As a comparison, our network achieve convergence more easily due to the use of advanced technologies such as SSTM and the encoder. The performance improvements are the result of combined optimization through the framework. The proposed framework is equivalent to other machine learning methods at very low SNRs, while at −10 dB its classification accuracy increases sharply. When SNR is below −10 dB, noise energy is much greater than signal energy. In this case, it is tough for all methods to extract the signal modulation submerged in the noise. And the system basically does not have modulation recognition capability, so the overall classification accuracy is close to 9.1%. It can be seen in Figure 8 that there is no significant performance improvement of VTCNN with SSTM. We consider this is in line with the results of previous research that training over frequency and sample rate mismatches does not have significant impact on the performance. SSTM has the effect of data normalization and stability improvement in our framework. The performance improvements lean on combined optimization through our framework. Overall classification accuracies of all methods at typical SNR are shown in Table 5.

### 5.2. Semi-Supervised Learning

An important contribution of the proposed GAN-based model is to provide the ability to learn under semi-supervised conditions. Thus, we compare our method with classical semi-supervised methods using different proportions of unlabeled samples. It is notable that three types of data exist in GAN’s semi-supervised learning training: labeled data, unlabeled data and generated data. When compared to other semi-supervised methods, we control the scale of the first two types of raw data.

We compare graph-based semi-supervised learning using a single learner, disagreement-based methods using multiple learners, and classic GAN. Graph-based semi-supervised learning [36] is a multiclass label propagation model that maps a given data set to a graph and spreads the labels by assessing the similarity among samples. Disagreement-based methods [37] use a co-training method with multiple learners. For multi-view data design, we trained two classifiers for IQ two-way data. One is the same CNN network as our C subnetwork. The other one is the LSTM network, which uses the information from the two views extracted by the two learners and effectively improve the generalization performance of the weak classifier. The GAN uses the classic D and G structure. The difference is that the classifier output is increased by one class; the extra class represents true or fake. This approach is used to check the tagged samples in the training set to determine whether the estimated tags are correct. That is, the calculation is classified as the corresponding probability, and for the unlabeled samples in the training set, the investigation involves judging whether the estimation is “true”. The probability of not being estimated as K + 1 is calculated. For the fake samples generated by the generator, this calculation determines whether the sample is estimated to be fake. That is, the probability of estimating the K + 1 class is calculated.

**Figure 9:**
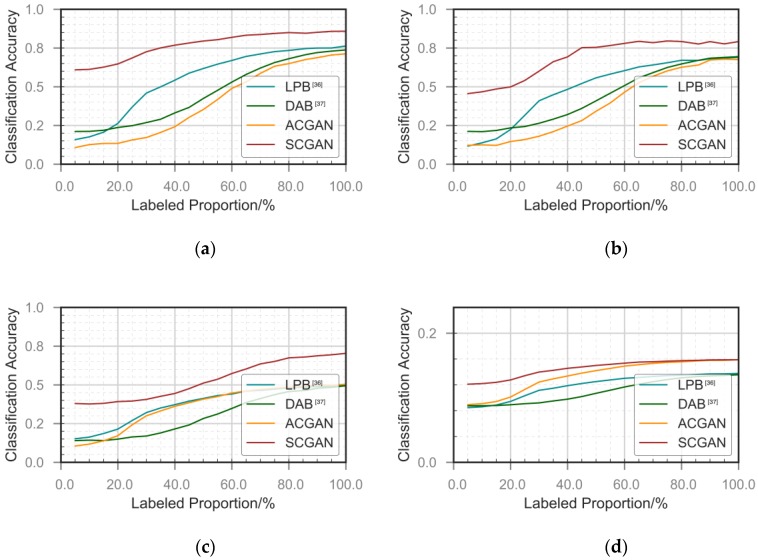
Classification accuracy correspond to: (**a**) 18 dB; (**b**) 5 dB; (**c**) −5 dB; and (**d**) −10 dB SNR.

We compare our approach to these three classic methods to show the effectiveness of semi-supervised learning. For the entire dataset, we consider the different proportions of supervised samples in the total amount of training examples, increasing from 10% to 90% by the rate of 5%. We verify the performance comparisons in these four cases. In the legend of Figure 9, LPB represents label propagation-based method [36]. DAB represents disagreement-based method [37]. We use various methods to benchmark the best classification performance achievable with all the labeled samples and test the various classifiers after training with the test dataset. The results are shown in Figure 9. 

When the percentage of labeled samples was less than the training data ratio, the classification results obtained by our network did not decrease significantly; however, the results of the graph-based and divergent methods declined significantly. We believe that the disadvantage of the graph-based approach is that when few labeled samples exist, the composition process merely take into account the training-sample set, which makes the position of the new sample in the graph is difficult to be known. When receiving a new sample or adding it to the original dataset, refactoring and remarking propagation leads to instability in the training process. The disagreement-based methods have few labeled samples, especially when the data distribution do not have multiple views. Performance comparison for semi-supervised methods in typical SNR is shown in Table 6.

### 5.3. Numerical Distinction

Fake samples generated by GAN framework have fooled both humans and machines into believing that they are indistinguishable from real samples. However, the radio signal samples generated by the GAN differ from the images but are also difficult to separate from the real samples visually. Under the principles of a zero-sum game, the generated data are close to the distribution pattern of training data, which improves the classification and discrimination capabilities of the network accordingly. Therefore, we use a qualitative method to verify whether the network can generate fake samples that meet the training data specifications. We evaluate the system attributes of the fake samples in two ways: from the characteristics of the radio signal and from the numerical characteristics of the samples. To evaluate the characteristics of the radio signal, generated samples are observed from the frequency domain and time domain. The modulation mode learned by the GANs through the original IQ signal are evaluated. 

**Figure 10:**
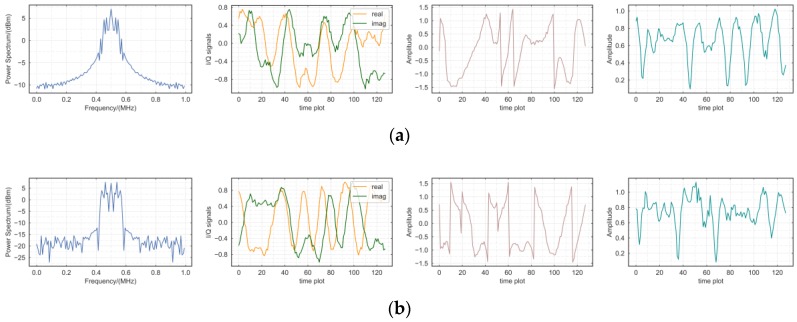

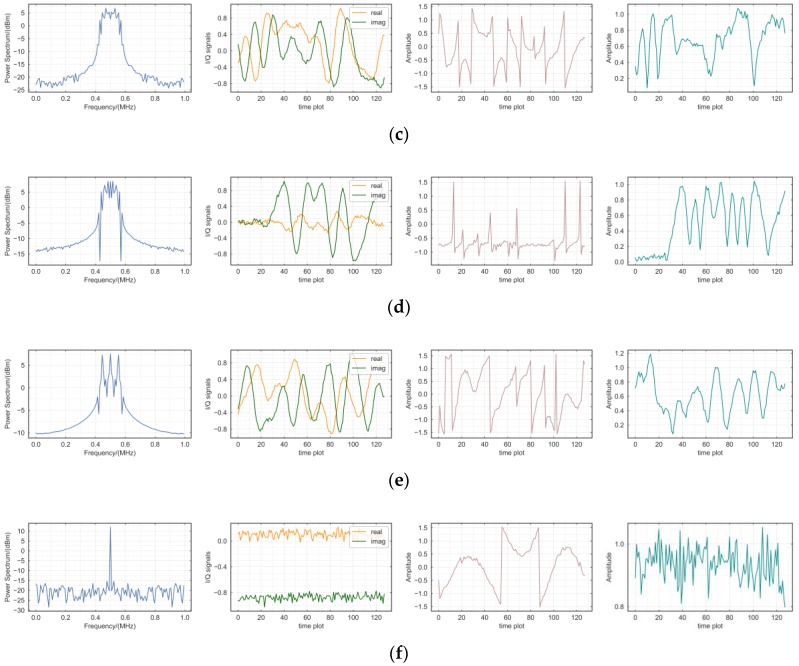
Power spectrum, I and Q, phase and amplitude signals time plot for various fake modulation signals: (**a**) 8PSK; (**b**) QPSK; (**c**) 16-QAM; (**d**) PAM4; (**e**) 64-QAM; (**f**) WBFM.

As shown in Figure 10, the fake samples generated by Signal Classifier Generative Adversarial Networks (SCGANs) have similar characteristics to the real radio signals in the frequency domain. In addition, it can be observed in time domain that the generated samples of different modulations have a significant degree of discriminations. This proves that our framework learns the modulation characteristics of signals.

Valle et al. proposed TequilaGAN [38], which is used to compare the characteristics of true and fake samples. This method compares real and fake samples by numerical analysis in the case where the discriminator and the human eye are unable to differentiate. They prove that the fake sample would violate the data specification learned from the real sample. We adapt this method to evaluate the real and fake samples.

**Figure 11:**
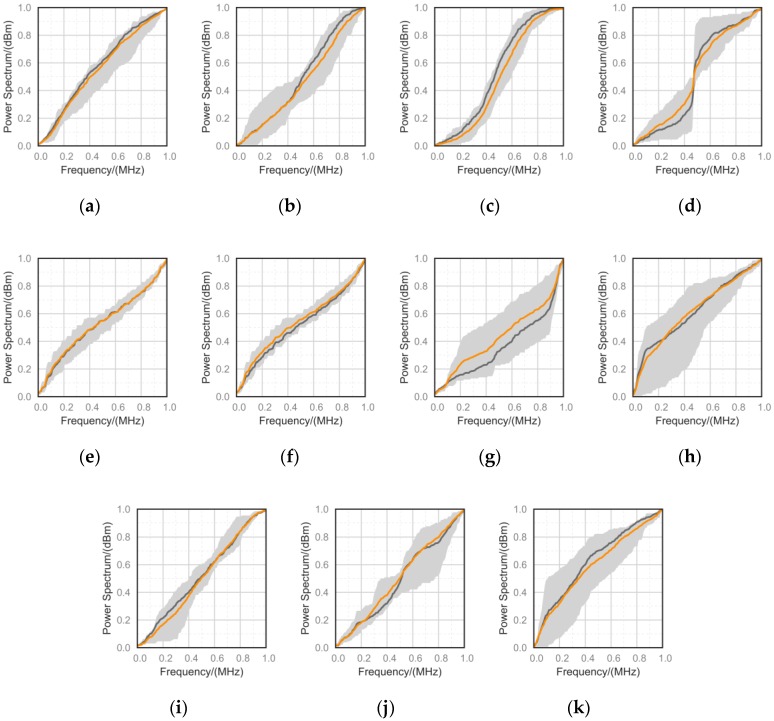
Empirical CDF computed on various fake modulation signals: (**a**) 8PSK; (**b**) AM-DSB; (**c**) AM-SSB; (**d**) BPSK; (**e**) QPSK; (**f**) CPFSK; (**g**) GFSK; (**h**) PAW4; (**i**) 16-QAM; (**j**) 64-QAM; (**k**) WBFM.

Figure 11 illustrates the empirical CDFs of the numerical distribution. We analyze the numerical properties of the generated samples by the method proposed by Valle et al. [38]. The generated samples satisfy the data distribution of different modulation types. Figure 11 shows that the samples generated by GAN are smooth and close to the distribution pattern, which differs between the training and test sets. The first hypothesis concerning the interpretation of the smooth approximation of the distribution pattern is that the network construction uses stochastic gradient descent and asymptotic convergence activation functions (such as sigmoid or tanh).

These two analysis methods demonstrate that the fake samples generated by the GAN meet the data specification and mimic the implicit characteristics of the radio data characteristics, since GAN learned the differences between modes with different modulation methods. The analysis explains why our approach surpasses similar deeper learning approaches.

## 6. Discussion and Conclusions 

In this paper, we introduced an innovative end-to-end learning framework for implementing radio signal identification tasks, which is an important facet of constructing the spectrum-sensing capability required by CS. The goal was to achieve feature extraction and learning from original sampled signals. Finally, we achieve a performance beyond that of similar deep learning methods. We used generational adversarial concepts by letting two independent neural networks undergo adversarial training. This approach does not require human assistance to recognize radio signals. In addition, to solve the problems that a GAN applied to radio signals is prone to mode collapse and nonconvergence, we proposed an advanced network design architecture that improves on the traditional GAN generation approach by adding an encoder, making it more suitable for radio tasks. We also separated the discriminator and classifier into individual networks and added the attention-based SSTM module before them. These methods enable our proposed GAN framework to achieve stable training and effectively avoid the adverse effects of model collapse on model performance. We verified the classification performance of the proposed network under supervised conditions. We also demonstrated that under semi-supervised conditions, the GAN learning model surpasses other semi-supervised learning methods, especially when only a small proportion of the training data are labeled. Our method achieves a significant performance improvement over other semi-supervised methods. Finally, we analyzed the numerical characteristics and signal characteristics of the GAN-generated samples and showed that our method is able to learn and mimic the characteristics of real radio signals. We believe that the significance of this capability far exceeds the modulation recognition task itself.

In future work, we plan to focus on exploring the interpretability of deep learning applied to radio signals and on the possibility of applying GAN to combining demodulation and decoding. We expect to achieve timing information recovery for raw physical-layer signals.

## Figures and Tables

**Table 1:** Proposed discriminator and classifier structure.

Layer Type	Input Size	Parameters	Activation Function
Convolution layer	2 × 128	1 × 3 filter kernel 32 feature maps	LeakyReLU
Pooling layer	2 × 128 × 32	1 × 2 average	None
Convolution layer	2 × 64 × 32	1 × 3 filter kernel 32 feature maps	LeakyReLU
Pooling layer	2 × 32 × 32	1 × 2 average	None
Dense layer 1	2048 × 2	2 neurons	Softmax
Dense layer 2	2048 × 11	11 neurons	Softmax

**Table 2:** Proposed encoder structure.

Layer Type	Input Size	Parameters	Activation Function
LSTM layer	2 × 128	10 kernel_size 10 filters	None
Flatten layer	2 × 128 × 10	None	None
Dense layer	256	100 neurons	ReLU

**Table 3:** Proposed generator structure.

Layer Type	Input Size	Parameters	Activation Function
Dense	256	8192 neurons	LeakyReLU
TransConvolution layer	2 × 128 × 32	1 × 3 filter kernel 256 feature maps	Tanh
TransConvolution layer	256 × 128 × 32	1 × 3 filter kernel 80 feature maps	Tanh
TransConvolution layer	80 × 128 × 32	1 × 3 filter kernel 1 feature maps	Tanh
Reshape layer	1 × 128 × 32	None	None

**Table 4:** Hyperparameters in SCGAN training.

Hyperparamenters	Value
Loss Function	Binary Crossentropy (D), Categorical Crossentropy (C)
Optimizer	RMSPROP (C, D), ADAM (G)
Initializer	Lecun Normal [29]
Learning Rate	0.0004 (C), 0.0001 (D, G)
Epochs	500
Mini Batch	256
Dropout Rate	0.5

**Table 5:** Overall classification accuracy versus typical SNR.

SNR (dB)	SVM_5	SVM_7	NBC	ACGAN	VTCNN	CNN_SSTM	SCGAN
−10	0.14	0.14	0.21	0.18	0.20	0.17	0.21
0	0.58	0.54	0.47	0.66	0.68	0.70	0.86
10	0.61	0.59	0.46	0.70	0.72	0.71	0.85

**Table 6:** Performance comparison for semi-supervised methods versus typical SNR.

Framework	SNR	Labeled PRO 10%	Labeled PRO 50%	Labeled PRO 90%
LPB [36]	High	0.14	0.56	0.68
	Medium	0.17	0.61	0.75
	Low	0.10	0.15	0.17
DAB [37]	High	0.21	0.40	0.69
	Medium	0.21	0.42	0.73
	Low	0.11	0.12	0.17
ACGAN	High	0.12	0.34	0.67
	Medium	0.12	0.35	0.70
	Low	0.11	0.17	0.19
SCGAN	High	0.46	0.75	0.77
	Medium	0.61	0.80	0.85
	Low	0.15	0.18	0.20
